# Black Patients Experience Higher Complication Burden and Distinct Valve Selection Patterns in Aortic Valve Replacement

**DOI:** 10.7759/cureus.102650

**Published:** 2026-01-30

**Authors:** Joshua D Greendyk, Julia DeLorenzo, Jonathan R Lopez, Afif Hossain, Abhishek Sharma

**Affiliations:** 1 Department of Medicine, Division of Cardiology, Rutgers University New Jersey Medical School, Newark, USA

**Keywords:** aortic stenosis (as), aortic valve replacment, racial disparity, ross procedure, surgical aortic valve replacement (savr), transcutaneous aortic valve replacement

## Abstract

Background

Young adults undergoing the Ross procedure for aortic valve replacement may have better outcomes than more conventional mechanical and bioprosthetic aortic valve replacement. This study evaluates racial disparities in access to the Ross procedure and postoperative outcomes, hypothesizing that Black patients may have differing Ross utilization due to constrained valve options, with persistent disparities in postoperative complications.

Methods

Cases of open aortic valve replacement (AVR) surgery between 2012 and 2021 from the American College of Surgeons National Surgical Quality Improvement Program (ACS-NSQIP) were isolated, with all transcatheter aortic valve replacement (TAVR) cases excluded. All data was deidentified with IRB exemption. Univariate analysis and propensity score matching with binary logistic regression were performed to assess the independent effect of black self-identified race on access and 30-day post-operative complications. Covariates included in the propensity models included age, gender, BMI, smoking status, as well as comorbidities including diabetes, congestive heart failure, kidney failure, and chronic obstructive pulmonary disease.

Results

Among 5,698 isolated AVR cases in ACS-NSQIP, most were White patients (93.7%), older (median age 71), and underwent mechanical AVR, with Ross procedures remaining rare at 260 (4.6%) cases. Black patients were less likely to receive mechanical AVR overall, but among those younger than 60, were more likely than White patients to undergo the Ross procedure. Across valve types, Black patients experienced higher rates of postoperative patient complications, including acute renal failure, prolonged mechanical ventilation, reintubation, cardiac arrest and 30-day mortality. An adjusted analysis demonstrated that Black race was an independent predictor of severe postoperative complications regardless of valve strategy.

Conclusions

Differences in AVR use and outcomes between Black and White patients reflect structural, socioeconomic, and clinical factors rather than biology. Higher Ross use among younger Black patients likely reflects greater comorbidity and limited valve options, while persistently worse outcomes highlight the impact of baseline risk and systemic inequities. Addressing these disparities requires research incorporating socioeconomic and care-delivery factors, alongside clinical strategies such as standardized referrals, preoperative optimization, and equity-focused decision support.

## Introduction

Globally, there is a growing demand for aortic valve replacement (AVR) [1]. The availability of multiple AVR techniques has led to continued debate over the most advantageous approach. Transcatheter aortic valve replacement (TAVR) has become the preferred method for AVR in older adults because of its superior outcomes when compared to surgical aortic valve replacement (SAVR), but the choice of treatment for younger and middle-aged patients, as well as elderly patients with comorbidities, is still controversial [2, 3]. Further research on surgical AVR is needed because mechanical, bioprosthetic, and Ross procedures remain the standard of care for younger and lower-risk patients, in whom TAVR use is limited by durability concerns and guideline recommendations.

While surgical AVR most commonly utilizes either bioprosthetic or mechanical valves, the less commonly performed Ross procedure is a method of AVR that utilizes the patient’s pulmonic valve to replace the aortic valve, followed by homograft replacement of the pulmonic valve [4,5]. For the Ross procedure, access disparities may relate to referral patterns, surgeon expertise, and institutional availability. Outcome disparities relate to baseline risk, perioperative care, and postoperative follow-up among patients who receive the Ross procedure. There is currently no clear consensus on which of the three procedures offers the best outcomes. While the overall survival rates for bioprosthetic and mechanical AVR are similar, bioprosthetic valve replacement offers a reduced risk of major bleeding when compared to mechanical valve replacement, but an increased likelihood of reoperation [6,7].

Current guidelines for AVR in patients with severe aortic stenosis indicate consideration of a mechanical AVR for patients younger than 60 years old and bioprosthetic AVR for patients older than 65 years old [8]. Though not currently recommended due to concerns over the lack of literature and the risk of valvular failure in either valve, recent studies have called for reconsideration of the Ross procedure with data indicating improved long-term survival, especially in younger patients, as well as improved stroke, bleeding, reintervention, and endocarditis outcomes when compared to mechanical and bioprosthetic AVR [9-14]. The current lack of consensus and literature on the use of the Ross procedure demands that more research be conducted on this method to determine if it may be a better alternative to mechanical and bioprosthetic AVR.

Minimally invasive techniques such as TAVR and the Ross procedure have transformed surgical interventions and improved patient outcomes, but the benefits of these improvements have not been recognized by all patient groups uniformly. While unequal treatment across racial groups has been recognized across many medical interventions, there is a paucity of research on the disparities that exist within minimally invasive procedures [15,16]. Inequities in healthcare exist not only amongst different racial groups but also across different ethnicities, genders, and weight classes. An estimated 85,000 AVR procedures are performed annually in the United States, but evidence suggests that access to these procedures may not be equal across racial and socioeconomic groups, with minorities facing a disadvantage [10,17]. The disparities experienced by Black patients have been postulated to multiple factors, including lower levels of physician trust among minority populations, reduced likelihood of referral for specialized procedures, lower insurance coverage rates, and poorer socioeconomic status [17]. Additionally, prior studies have suggested that these disparities may be partly explained by a significantly lower risk of developing severe aortic stenosis among African American patients compared with Caucasian patients [17].

With the renewal of interest in the Ross procedure, it is important to assess access and patient outcomes across patient groups receiving this treatment to recognize existing disparities. The present study aims to examine the influence of race on access and post-surgical outcomes in patients undergoing bioprosthetic, mechanical or Ross procedure aortic valve replacement utilizing a database with participation from governmental, municipal, academic, and community hospitals. We hypothesized that Black patients may have differing Ross utilization due to constrained valve options, with persistent disparities in postoperative complications. 

This article was previously presented as a meeting abstract at the 2024 American College of Cardiology Annual Meeting in Atlanta, Georgia on April 6, 2024.

## Materials and methods

Data acquisition

The American College of Surgeons National Surgical Quality Improvement Program (ACS-NSQIP) is a nationwide, multicenter database that collects pre-operative, intra-operative, and 30-day postoperative data on a randomly selected sample of patients. All data were deidentified with an IRB exemption. The statistical analyses, interpretations, and conclusions presented are solely those of the authors and have not been verified or endorsed by the ACS-NSQIP or the participating hospitals.

Patients who had undergone aortic valve replacement between 2012 and 2021 were identified and included in the study using Current Procedural Terminology (CPT) codes encompassing both conventional approaches (i.e. mechanical aortic valve replacement, bioprosthetic aortic valve replacement), compared to the Ross procedure. Mechanical and bioprosthetic valves were grouped as a single conventional cohort because they share similar operative exposure and perioperative risk profiles compared with the Ross procedure. Mechanical aortic valve replacement was identified using CPT codes 33405, 33406, and 33410. Bioprosthetic aortic valve replacement was identified using CPT codes 33361, 33362, 33363, 33364, 33365, and 33366. The Ross procedure was identified using CPT codes 33411, 33412, 33413, 33414, 33415, and 33440. Only isolated, primary surgical AVR cases were included. Redo operations, concomitant cardiac procedures, and emergent cases were excluded to reduce confounding from surgical complexity. Missing data were handled using complete-case analysis. Race reporting completeness was assessed, and cases with missing race data were excluded. The ACS-NSQIP does not include data on hospital-level clustering, surgeon volume or center volume.

Variables of interest

Demographic variables assessed include patient self-identified race (White or Black), age greater than 60 years, and sex. Preoperative comorbidities summarized within this analysis include ASA class greater than two, current or past smoking history within one year, diabetes, congestive heart failure, medicated hypertension, kidney failure, steroid use for a chronic disease, current bleeding disorder, and current diagnosis of COPD. Complications were compiled into any, minor and severe complication groups as previously described [18,19]. Incidence of postoperative complications included any complications (superficial incisional surgical site infection (SSI), deep incisional SSI, organ space SSI, wound disruption, pneumonia, unplanned intubation, pulmonary embolism (PE), failure to wean off the ventilator within 48 hours, progressive renal insufficiency, acute renal failure, urinary tract infection (UTI), ischemic stroke or cerebrovascular accident (CVA), cardiac arrest, myocardial infarction (MI), deep vein thrombosis (DVT), return to the operating room, unplanned readmission, and/or systemic sepsis, and death); minor complications (UTI, superficial surgical site infection, pneumonia, and unplanned readmission); severe complications (sepsis, septic shock, MI, cardiac arrest, deep wound infection, deep surgical site infection, organ/space surgical site infection, wound dehiscence, PE, DVT, progressive renal insufficiency, renal failure, CVA, transfusion, unplanned reintubation, failure to wean off the ventilator within 48 hours, 30-day mortality (death), unplanned readmission and unplanned reoperation). The primary outcome was the utilization of the Ross procedure according to patient-reported race, categorized as White patients vs. Black patients. Secondary outcomes were the rates of any, minor, and severe complications, stratified by patient race. Postoperative complications were assessed for 30 days from the time of aortic valve replacement. Primary analyses evaluated differences in procedural utilization and 30-day mortality. Secondary analyses assessed major postoperative complications. Additional exploratory analyses examined individual complication subtypes and resource utilization.

We assessed whether postoperative outcomes differed between Black patients and White patients within the Ross procedure subgroup compared with those undergoing conventional aortic valve replacement. To accomplish this, complication analyses were stratified by procedural type, with mechanical and bioprosthetic valve replacements grouped as the conventional approach and Ross procedures analyzed as a distinct cohort. The objectives of this study were intentionally divided to distinguish upstream disparities in procedural access from downstream differences in postoperative outcomes among patients undergoing surgical aortic valve replacement. First, we sought to evaluate racial differences in access to and utilization of surgical AVR techniques, including bioprosthetic, mechanical, and Ross procedures, as a measure of procedural selection and access. Second, among patients who underwent surgical AVR, we examined racial differences in short-term postoperative outcomes, including 30-day mortality, complications, and length of stay. This separation was designed to clarify whether observed disparities reflect differences in treatment selection versus differences in perioperative risk and postoperative care.

Propensity score-matching

Given the aim of this paper, we sought to isolate race as an independent predictor of postoperative outcomes while all other clinical variables are matched. Demographic characteristics and comorbidities stratified by self-identified race are summarized and compared, and these variables were used in a subsequent propensity score-matched analysis between Black patients and White patients. Propensity score-matching was performed using a nearest-neighbour matching algorithm with a 1:1 ratio to reduce baseline differences between Black patients and White patients undergoing aortic valve replacement, balancing for all available clinical variables except for self-identified race. Calliper width was set at 0.2 standard deviations of the propensity score. Propensity matching balance was evaluated using standardized mean differences, with thresholds <0.1 indicating adequate covariate balance.

Statistical analysis

Rates of Ross procedure utilisation were computed and compared across clinicodemographic categories. Pearson’s χ2 or Fisher’s exact tests and one-way ANOVA were used to analyze categorical and continuous variables, respectively. Variables significantly associated with race on univariate analysis (p≤0.05) were entered into binary logistic regressions to identify the independent predictors of race on demographics and clinical outcomes. Model fit was assessed using a likelihood ratio test comparing the final model with the intercept-only model. Model explanatory power was summarized using pseudo R² statistics. p-values ≤0.05 were considered statistically significant; all tests were two-sided. Statistical analyses were performed using IBM SPSS Statistics for Macintosh, version 26 (IBM Corp., Armonk, NY, USA).

## Results

Patient clinical demographics

Table 1 summarizes patient demographics and clinical comorbidities. The initial query returned 5,698 cases of AVR, which comprised 5,339 (93.7%) White patients and 359 (6.3%) Black patients (Table 1). Median patient age was 71 (IQR 61-78), and 2,021 (35.5%) were female. Most patients underwent mechanical AVR for a total of 4,482 (78.7%), while only 956 (16.8%) and 260 (4.6%) underwent bioprosthetic or Ross procedure, respectively. Black patients were significantly less likely to undergo mechanical AVR than White patients, although Black patients <60 years of age were significantly more likely to undergo Ross procedure than White patients <60 years of age. Age was modelled as a binary variable (>60 years) to align with guideline-based risk stratification and valve-selection thresholds, and to improve interpretability of effect estimates. Black patients were significantly less likely to have hypoalbuminemia (albumin <3.4 g/dL) and more likely to have a history of smoking. Black patients were more likely to suffer from congestive heart failure (CHF), have medicated hypertension, kidney failure, and have a history of transfusion compared to White patients (Table 1). Significant demographics and comorbidities were used in subsequent regression models.

**Table 1:** Univariate analysis and multivariate logistic regression of the association of self-identified race with surgical approach and preoperative comorbidities for the entire cohort. The data has been represented as n (%). p-values are considered significant at <0.05 using Pearson’s χ2. BMI: body mass index; ASA: American Society of Anesthesiologists Score; dep. DM: dependent diabetes mellitus; CHF: congestive heart failure; COPD: chronic obstructive pulmonary disease; CI: confidence interval.

Variables	White Patients, N=5339, n (%)	Black Patients, N=359, n (%)	Test Value (χ2)	p-value	Odds Ratio	95% CI
							Mechanical AVR	4214 (78.9%)	268 (74.7%)	3.67	0.056	-	-
Bioprosthetic AVR	900 (16.9%)	56 (15.6%)	0.38	0.537	-	-
Ross Procedure	225 (4.2%)	35 (9.7%)	23.67	<0.001	-	-
Age > 60 years	4197 (78.6%)	198 (55.2%)	104.94	<0.001	0.355	0.272-0.437
Male Gender	3461 (64.8%)	216 (60.2%)	3.19	0.074	-	-
BMI >30	2392 (44.9%)	144 (40.3%)	2.88	0.090	-	-
ASA > 2	5262 (99.5%)	353 (99.7%)	0.40	0.529	-	-
Albumin <3.4	3957 (74.1%)	223 (62.1%)	24.88	<0.001	0.631	0.500-0.797
Smoker	715 (13.4%)	96 (26.7%)	49.10	<0.001	1.885	1.444-2.459
Insulin dep. DM	378 (10.7%)	36 (13.8%)	2.31	0.129	-	-
Non-insulin dep. DM	1503 (28.2%)	114 (31.8%)	2.15	0.143	-	-
CHF	999 (18.7%)	114 (31.8%)	36.41	<0.001	1.657	1.295-2.121
Hypertension	4103 (76.8%)	309 (86.1%)	16.37	<0.001	2.511	1.821-3.463
Kidney Failure	118 (2.2%)	51 (14.2%)	168.20	<0.001	5.01	3.447-7.282
Steroid Use	194 (3.6%)	17 (4.7%)	1.15	0.285	-	-
Bleeding Disorder	482 (9.0%)	27 (7.5%)	0.94	0.333	-	-
COPD	552 (10.3%)	48 (13.4%)	3.08	0.070	-	-
Transfusion	125 (2.3%)	15 (4.2%)	4.74	0.030	1.064	0.586-1.931

A multivariable analysis was performed to determine the independent association of self-identified race with clinical characteristics (Table 1). Separate models were fit for each covariate that was significant on univariate analysis to derive odd ratios, which showed that Black patients who underwent AVR were less likely to be over age 60 (OR 0.355, 95% CI, 0.272-0.437, p<0.001), have hypoalbuminemia (albumin <3.4 g/dL) (OR 0.631, 95% CI 0.5-0.797, p<0.001). Patients self-reported as Black patients were more likely to be smokers (OR 1.885, 95% CI 1.444-2.459, p=0.002), have a history of CHF (OR 1.657, 95% CI 1.295-2.121, p=0.003), hypertension (OR 2.511, 95% CI 1.821-3.463, p<0.001), and kidney failure (OR 5.01, 95% CI 3.447-7.282, p<0.001) (Table 1). 

Association of race/ethnicity and rates of conventional approach complications

Table 2 summarizes operative course and surgical outcomes for the cohort that received either mechanical or bioprosthetic AVR. Patients self-reported as Black patients were significantly more likely to have longer operation times, experience a longer total length of stay, and be re-admitted within 30 days. Interestingly, postoperative length of stay was not significantly different, whereas total length of stay was longer and statistically significant, likely reflecting delayed time to operation rather than differences in postoperative recovery. Black patients were also more likely to experience higher rates of reintubation, cardiac arrest, deep vein thrombosis (DVT), acute renal failure (ARF), be mechanically ventilated for greater than 48 hours, and 30-day mortality. Black patients also had higher rates of any complication, minor complication, and severe complication (Table 2).

**Table 2:** Univariate analysis of the association of self-reported race on operative course and surgical complications for patients who underwent conventional approach (i.e. mechanical and bioprosthetic AVR). The data has been represented as n (%) or mean ± SD. p-values significant at <0.05 using one-way ANOVA and Pearson’s χ2 or *Fisher's exact test when expected value ≤5. a: any complication, b: minor complication, c: severe complication. AVR: aortic valve replacement; LOS: length of stay; SSI: surgical site infection; PE: pulmonary embolism; UTI:  urinary tract infection; DVT: deep vein thrombosis; CVA: cerebral vascular accident; MI: myocardial infarction; ARF: acute renal failure.

Variables	White Patients, N=5114, n (%)		Black Patients, N=324, n (%)		Test Value	p-value	
Operative Time	247 ± 119		277 ± 128		23.05	<0.001	
Post-Op LOS	6.58 ± 10.7		7.07 ± 15.1		1.56	0.439	
Total LOS	8.08 ± 11.9		11.19 ± 26.1		21.95	<0.001	
Superficial SSI ^a, b^	75 (1.5%)		2 (0.6%)*		-	0.154	
Deep SSI ^a, c^	10 (0.2%)		2 (0.6%)*		-	0.158	
Organ Space SSI ^a, c^	16 (0.3%)		3 (0.9%)*		-	0.100	
Dehiscence ^a, c^	19 (0.4%)		0 (0.0%)*		-	0.311	
Pneumonia ^a, b, c^	274 (5.4%)		25 (7.7%)		3.26	0.071	
Reintubation ^a, c^	213 (4.2%)		22 (6.8%)		5.08	0.024	
PE ^a, c^	22 (0.4%)		0 (0.0%)*		-	0.237	
UTI ^a, b^	77 (1.5%)		8 (2.5%)		1.84	0.175	
Cardiac Arrest ^a, c^	156 (3.1%)		18 (5.6%)		6.17	0.013	
DVT ^a, c^	51 (1.0%)		9 (2.8%)		8.82	0.003	
Systemic Sepsis ^a, c^	83 (1.6%)		5 (1.5%)*		-	0.912	
Septic Shock ^a, c^	75 (1.5%)		8 (2.5%)		2.04	0.120	
CVA ^a, c^	111 (2.2%)		6 (1.9%)		0.15	0.701	
MI ^a, c^	37 (0.7%)		3 (0.9%)*		-	0.429	
ARF ^a, c^	112 (2.2%)		13 (4.0%)		4.51	0.034	
Vent >48 hours ^a, c^	336 (6.6%)		39 (12%)		14.18	<0.001	
Any Complication ^a, c^	1415 (27.7%)		117 (36.1%)		10.73	0.002	
Minor Complication ^a, c^	799 (15.6%)		70 (21.6%)		8.12	0.004	
Severe Complication ^a, c^	1400 (27.4%)		125 (38.6%)		18.96	<0.001	
30-Day Mortality^ a, c^	204 (4.0%)		23 (7.1%)		7.37	0.007	
Re-operation^ a, c^	435 (8.5%)		34 (10.5%)		1.53	0.216	
30-Day Readmission ^a, b, c^	482 (9.4%)		45 (13.9%)		6.94	0.008	

Table 3 summarizes the logistic regression evaluating the effect of race on patient outcomes in those who received either mechanical or bioprosthetic AVR. Clustering by hospital or year was not accounted for in the regression. Self-reported as Black patients (adjusted for age >60, albumin <3.4, smoking status, congestive heart failure, hypertension, and kidney failure) was shown to be an independent predictor of cardiac arrest (OR=1.717, 95% CI 1.013-2.908), DVT (OR=2.856, 95% CI 1.321-6.172), mechanical ventilation for greater than 48 hours (OR=1.551, 95% CI 1.067-2.253) and severe complication (OR=1.377, 95% CI 1.069-1.773) for AVR patients undergoing mechanical or bioprosthetic AVR (Table 3).

**Table 3:** Binary logistic regression of self-reported race on postoperative outcomes in patients who underwent conventional approach (i.e. mechanical and bioprosthetic AVR) * adjusted for age >60, albumin <3.4, smoking status, congestive heart failure, hypertension, and kidney failure. 95% confidence intervals (CI) are significant if the range does not include 1. DVT: deep vein thrombosis; ARF: acute renal failure; OR: odds ratio; CI: confidence interval.

Variables	Adjusted OR*	95% CI	p-value
Reintubation	1.415	0.875-2.287	0.157
Cardiac Arrest	1.717	1.013-2.908	0.045
DVT	2.856	1.321-6.172	0.008
ARF	1.733	0.941-3.193	0.078
Vent >48 hrs	1.551	1.067-2.253	0.021
Any Complication	1.234	0.964-1.581	0.095
Minor Complication	1.226	0.916-1.640	0.17
Severe Complication	1.377	1.069-1.773	0.013
30-Day Mortality	1.564	0.969-2.524	0.067
30-Day Readmission	1.703	0.893-3.248	0.106

Association of race/ethnicity and rates of Ross procedure complications

Table 4 summarizes operative course and surgical outcomes for the cohort that received the Ross procedure. Statistical power was limited by the small Black Ross cohort. Black patients were found to have a significantly longer total length of hospital stay after receiving AVR with the Ross procedure. However, their postoperative length of stay was not significantly different, which may be due to a longer preoperative hospital course. Neither univariate analysis nor binary logistic regression yielded any other significant differences between rates of complications after undergoing the Ross procedure when stratified by self-reported White patients vs. Black patients (Tables 4, 5). Confidence intervals were wider for the Ross procedure group due to the low number of cases, suggesting potential model instability and overfitting. Interaction testing between race and age or comorbidity was not explored.

**Table 4:** Univariate analysis of the association of self-reported race on operative course and surgical complications for patients who underwent Ross procedure. The data has been represented as n (%) or mean ± SD. p-values significant at <0.05 using one-way ANOVA, Pearson’s χ2 or *Fisher's exact test when expected value ≤5. a: any complication, b: minor complication, c: severe complication. AVR: aortic valve replacement; LOS: length of stay; SSI: surgical site infection; PE: pulmonary embolism; UTI: urinary tract infection; DVT: deep vein thrombosis; CVA: cerebral vascular accident; MI: myocardial infarction; ARF: acute renal failure.

Variables	White Patients, N=225, n (%)		Black Patients, N=35, n (%)		Test Value	p-value	
Operative Time	296 ± 125		315 ± 134		0.67	0.412	
Post-Op LOS	6.29 ± 13.9		9.74 ± 6.8		2.07	0.152	
Total LOS	8.36 ± 15.3		14.17 ± 10.8		4.72	0.031	
Superficial SSI ^a, b^	1 (0.4%)		0 (0.0%)*		-	0.865	
Organ Space SSI ^a, c^	4 (1.8%)		0 (0.0%)*		-	0.559	
Dehiscence ^a, c^	3 (1.3%)		0 (0.0%)*		-	0.647	
Pneumonia ^a, b, c^	13 (5.8%)		2 (5.7%)*		-	0.673	
Reintubation ^a, c^	9 (4.0%)		2 (5.7%)*		-	0.45	
UTI ^a, b^	6 (2.7%)		0 (0.0%)*		-	0.416	
Cardiac Arrest ^a, c^	10 (4.4%)		2 (5.7%)*		-	0.498	
DVT ^a, c^	4 (1.8%)		1 (2.9%)*		-	0.518	
Systemic Sepsis ^a, c^	5 (2.2%)		2 (5.7%)*		-	0.24	
Septic Shock ^a, c^	5 (2.2%)		0 (0.0%)*		-	0.482	
CVA ^a, c^	6 (2.7%)		0 (0.0%)*		-	0.416	
MI ^a, c^	1 (0.4%)		0 (0.0%)*		-	0.865	
ARF ^a, c^	7 (3.1%)		0 (0.0%)*		-	0.359	
Vent >48 hours ^a, c^	12 (5.3%)		2 (5.7%)*		-	0.587	
Any Complication ^a, c^	68 (30.2%)		10 (28.6%)		0.04	0.843	
Minor Complication ^a, c^	38 (16.9%)		4 (11.4%)*		-	0.414	
Severe Complication ^a, c^	74 (32.9%)		10 (28.6%)		0.26	0.611	
30-Day Mortality^ a, c^	21 (9.3%)		1 (2.9%)*		-	0.171	
Re-operation^ a, c^	18 (8.0%)		3 (8.6%)*		-	0.559	
30-Day Readmission ^a, b, c^	22 (9.8%)		3 (8.6%)*		-	0.558	

**Table 5:** Binary logistic regression of self-reported race on postoperative outcomes in patients who underwent Ross procedure. * adjusted for age >60, albumin <3.4, smoking status, congestive heart failure, hypertension, and kidney failure. 95% confidence intervals (CI) significant if range does not include 1. DVT: deep vein thrombosis; ARF: acute renal failure; OR: odds ratio; CI: confidence interval.

Variable	Adjusted OR*	95% CI	p-value
Reintubation	1.821	0.356-9.321	0.472
Cardiac Arrest	1.608	0.311-8.316	0.572
DVT	1.571	0.163-15.12	0.696
Vent >48 hours	1.428	0.293-6.962	0.659
Any Complication	1.266	0.527-2.855	0.636
Minor Complication	0.764	0.243-2.397	0.644
Severe Complication	1.201	0.517-2.791	0.671
30-Day Mortality	0.358	0.045-2.862	0.333
30-Day Readmission	1.031	0.237-3.889	0.964

Propensity score-matching analysis

A propensity score-matching algorithm with a 1:1 ratio was utilized, yielding 350 patients of White self-reported race and 359 patients of Black self-reported race, including cases from all three AVR techniques. Race was modelled as the dependent variable to specifically assess differences in procedural selection and postoperative outcomes associated with self-reported race while accounting for baseline covariates. This approach allows interpretation of how observed characteristics, rather than surgical technique or outcomes themselves, relate to disparities in care. Logistic regression demonstrated that Black patients were less likely to undergo bioprosthetic repair compared to mechanical (OR 0.351, 95% CI 0.244-0.506). Logistic regression of Black self-reported race on postoperative complications found that among this cohort, Black patients were more likely to suffer any complications (OR 1.604, 95% CI 1.112-2.315), minor complications (OR 1.498, 95% CI 1.014-2.212), and severe complications (OR 1.654, 95% CI 1.203-2.221) (Table 6). It is important to note that residual confounding from unmeasured socioeconomic factors, hospital-level characteristics, and other structural determinants may contribute to these observed associations and should be considered when interpreting the results.

**Table 6:** Logistic regression of black self-reported race on surgical approach and postoperative complications within propensity score-matched cohorts. * Compared to mechanical AVR. 95% confidence intervals (CI) significant if range does not include 1. OR: odds ratio; CI: confidence interval.

Variables	OR		95% CI		p-value
Bioprosthetic AVR*	0.351		0.244-0.506		<0.001
Ross Procedure*	1.071		0.621-1.846		0.805
Any	1.604		1.112-2.315		0.012
Minor	1.498		1.014-2.212		0.042
Severe	1.654		1.203-2.274		0.002
Unplanned Readmission	1.398		0.880-2.221		0.156
Unplanned Re-operation	1.28		0.769-2.131		0.343

## Discussion

The principal findings of the present investigation are as follows: Black patients under the age of 60 were significantly more likely to undergo the Ross procedure than their White counterparts; Black race was associated with higher resource utilization (longer operation time, longer total length of stay, higher rates of readmission, reintubation, and mechanical ventilation); propensity score-matched analysis demonstrates that Black patients were more likely to experience any complications, minor complications and severe complications; Black patients had higher rates of death within 30 days of surgery. However, adjusted analyses suggest that higher 30-day mortality did not reach statistical significance.

These findings taken together suggest that racial differences in procedural selection and postoperative outcomes among patients undergoing AVR are occurring within a markedly unequal baseline clinical context. Of note, TAVR data are absent, limiting inferences about overall AVR access and selection. Black patients in this cohort presented with a substantially higher burden of cardiovascular and systemic comorbidities, including congestive heart failure, hypertension, chronic kidney failure, and active smoking. This is consistent with Black patients in many national surgical datasets [20]. These conditions are independently associated with increased operative complexity, prolonged recovery, and higher complication risk [21]. The higher prevalence of kidney failure in particular offers a clinically plausible explanation for the lower utilization of mechanical valves and the relatively higher use of the Ross procedure among younger Black patients, as concerns regarding lifelong anticoagulation may influence surgical decision-making [22]. Additionally, longer operative times could reflect case complexity or centre-level factors not captured in ACS-NSQIP. In this context, procedural selection may reflect risk mitigation rather than preferential access to advanced interventions.

Black patients also experienced higher rates of postoperative complications, including longer operative times, extended hospitalizations, higher readmission rates, and increased frequencies of both minor and severe complications, including cardiac arrest, prolonged mechanical ventilation, thromboembolic events, and acute renal failure. However, there is the chance that self-reported race may involve misclassification or heterogeneity, affecting results. Although multivariable and propensity score-matched analyses demonstrated that self-identified Black race remained associated with higher odds of severe complications even after adjustment for several comorbid factors, these associations may reflect residual confounding from unmeasured variables such as disease severity at presentation, frailty, access to perioperative resources, hospital and surgeon volume, and structural barriers to care [23]. Importantly, the persistence of worse outcomes despite adjustment should therefore be interpreted as signalling the cumulative impact of systemic and clinical inequities rather than a biological effect of race.

The worst short-term outcomes observed among Black patients appear to arise from a complex, interrelated interplay between race, valve selection, and underlying socioeconomic and clinical factors rather than from procedural choice alone. Socioeconomic factors not captured in NSQIP, such as insurance status, delayed referral, limited access to high-volume centres, and reduced availability of preoperative optimization, likely compound clinical risk by increasing disease severity at presentation and limiting perioperative resources [24]. Thus, race functions less as a biologic determinant and more as a marker for cumulative exposure to structural disadvantage, comorbidity burden, and constrained clinical decision-making. The convergence of higher baseline risk, restricted valve options, and systemic inequities likely explains why Black patients experience worse short-term outcomes.

The Ross procedure remains underutilized due to concerns about technical complexity and the risk of reoperation on both aortic and pulmonary valves [25]. While various healthcare services encounter potential barriers to accessibility, high-tech procedures face heightened susceptibility to unequal utilization, particularly among marginalized populations. The disparate availability of such technologies can perpetuate structural racism within the medical field, characterized by systemic biases across societal institutions, ideologies, and processes that exacerbate disparities among racial and ethnic groups [26]. Preferential access to healthcare services by privileged racial and ethnic groups contributes to disparities in health outcomes and quality of life. Racial and ethnic segregation in metropolitan areas may concentrate vulnerable populations in impoverished areas, where limited public investment can impede access to care despite proximity to major medical centres [27].

Addressing the disparities identified in this analysis will require multifaceted, system-level interventions that extend beyond operative technique alone. First, standardized and transparent referral pathways for aortic valve disease, particularly for younger patients who may be candidates for specialized procedures such as the Ross operation, could help reduce variability in access and ensure timely evaluation at experienced centres. Second, targeted patient education initiatives focused on valve options, risks, and long-term implications may empower patients to engage more fully in shared decision-making, particularly in populations that have historically experienced lower levels of trust and information asymmetry within the healthcare system. This is paramount due to recent technological advances that can affect outcomes [28]. Third, institutional efforts to address implicit bias through structured training and decision-support tools may help mitigate unconscious influences on procedural selection and perioperative management. Finally, greater emphasis on preoperative risk stratification and optimization of comorbid conditions, including hypertension, renal dysfunction, and nutritional status, coupled with increased referral to high-volume surgeons and centres when feasible, may improve short-term outcomes for high-risk patients [29].

To better understand and address these disparities, future studies should directly operationalize and measure structural and socioeconomic factors. Patient-level data such as income, education, insurance status, and neighbourhood-level social determinants of health should be collected. Referral patterns, distance to high-volume centres, and timing of evaluation can quantify access disparities. Centre and surgeon-level characteristics, including procedural volume, staffing, and resources, should also be incorporated. Metrics of patient engagement, health literacy, and trust in the healthcare system could capture informational inequities. Using prospective registries or linked administrative datasets to collect these variables would allow direct measurement of structural drivers rather than relying on inferred associations. Collectively, these strategies underscore the need to shift from race-focused interpretations toward equity-driven care models that address the structural, clinical, and informational factors underlying disparities in aortic valve disease management.

Limitations

This study is subject to certain limitations. The ACS-NSQIP includes race as self-reported and therefore reflects societal categories and is not necessarily aligned with ancestry. Additionally, there is the potential for misclassification of procedural type based on CPT coding. Missing data were handled using complete-case analysis. The analysis includes only patients who underwent surgery, introducing potential survivorship and selection bias. Race reporting completeness was assessed, and cases with missing race data were excluded. The fact that the study spanned almost 10 years may have introduced temporal changes in trends that could have influenced access or outcomes. The ACS-NSQIP does not include data on hospital-level clustering, surgeon volume or centre volume. In the data obtained from ACS-NSQIP, there was a significantly higher number of White patients compared to the Black patients who received AVR. Race was dichotomized into White and Black categories to allow for adequate statistical power and stable estimates, given the relatively small representation of other racial and ethnic groups within the ACS-NSQIP dataset for patients undergoing aortic valve replacement. However, by limiting the analysis to White patients and Black patients, the findings may not be applicable to patients of other racial or ethnic backgrounds, including Hispanic, Asian, Native American, or multiracial populations, who may experience distinct patterns of access, referral, and surgical outcomes.

Additionally, dichotomization risks oversimplifying race as a binary construct and may obscure heterogeneity within racial groups, as well as the influence of intersecting social, cultural, and structural determinants of health. Therefore, dichotomizing continuous variables for adjustment could introduce residual confounding. Another limitation of this analysis is the inability to account for key unmeasured confounders, including socioeconomic status, insurance type, hospital and surgeon procedural volume, and geographic variation. These factors are well established drivers of access to specialized cardiac procedures and postoperative outcomes and are not fully captured within the ACS-NSQIP database. Consequently, these unmeasured variables may explain a proportion of the observed disparities. Although propensity matching and multivariable adjustment were used to balance observed covariates, these methods cannot account for unmeasured or imperfectly captured variables. As a result, observed associations should not be interpreted as causal, and the contribution of structural factors may be underestimated. Additionally, the Ross procedure subgroup analysis is severely underpowered, making the findings from this subgroup hypothesis-generating rather than conclusive. The ACS-NSQIP only offers a cross-sectional perspective regarding patient care; true preoperative surgical indications are not available, and operative outcomes past 30 days cannot be assessed. Finally, as the ACS-NSQIP represents a national sample of patients, these findings are descriptive and not generalizable to specific healthcare regions or institutions.

## Conclusions

This analysis demonstrates that differences in procedural utilization and short-term outcomes between Black patients and White patients undergoing AVR are unlikely to reflect biologic effects of race and instead point to the cumulative impact of structural, socioeconomic, and clinical factors that shape access, valve selection, and perioperative risk. Although younger Black patients were more likely to undergo the Ross procedure, this finding must be interpreted within the context of higher comorbidity burden and constrained valve options. The persistently higher rates of 30-day mortality and postoperative complications underscore how baseline risk, delayed or fragmented care, and systemic inequities may outweigh procedural choice in determining outcomes. However, adjusted analyses suggest higher 30-day mortality may not truly reach statistical significance. In this study, findings postulate differences in AVR utilization and short-term outcomes by race. However, observed differences may reflect associations within the limitations of the database and causal mechanisms; therefore cannot be inferred.

Future efforts should focus on disentangling these structural drivers through studies incorporating socioeconomic variables, referral patterns, hospital and surgeon volume, and disease severity at presentation. Clinically, implementation of standardized referral pathways, enhanced preoperative optimization, and equity-focused decision support may represent pragmatic steps toward reducing disparities and improving outcomes for historically underserved populations undergoing aortic valve replacement. These recommendations apply primarily to surgical AVR, though some principles may extend to broader aortic valve care pathways. Future studies could use detailed socioeconomic and referral data, prospective designs to clarify structural drivers and analyses incorporating hospital and surgeon volume, comorbidity burden, and disease severity to help address unanswered questions.
